# No differences in clinical outcomes with the addition of viral load testing to CD4 cell count monitoring among HIV infected participants receiving ART in rural Uganda: Long-term results from the Home Based AIDS Care Project

**DOI:** 10.1186/s12889-016-2781-y

**Published:** 2016-02-01

**Authors:** Stephen Okoboi, Paul John Ekwaru, James D. Campbell, Aggrey Egessa, Racheal King, Celestin Bakanda, Emmy Muramuzi, Frank Kaharuza, Samuel Malamba, David M. Moore

**Affiliations:** 1 The AIDS Support Organization-TASO, Headquarters, Mulago Hospital Complex, P.O BOX 10443, Kampala, Uganda; 2 University of Alberta, Edmonton, Canada; 3 University of Maryland School of Medicine, Baltimore, MD USA; 4 University of California, San Francisco, USA; 5 Makerere University School of Public Health, Kampala, Uganda; 6 BC Centre for Excellence in HIV/AIDS, Vancouver, Canada; 7 University of British Columbia, Vancouver, Canada

**Keywords:** Antiretroviral therapy, Virologic failure, Morbidity, Mortality, Sub-Saharan Africa, Uganda

## Abstract

**Background:**

We compared clinical outcomes among HIV-infected participants receiving ART who were randomized to viral load (VL) and CD4 cell count monitoring in comparison to CD4 cell count monitoring alone in Tororo, Uganda.

**Methods:**

Beginning in May 2003, participants with CD4 cell counts <250 cells/μL or WHO stage 3 or 4 disease were randomized to clinical monitoring alone, clinical monitoring plus quarterly CD4 cell counts (CD4-only); or clinical monitoring, quarterly CD4 cell counts and quarterly VL testing (CD4-VL). In 2007, individuals in clinical monitoring arm were re-randomized to the other two arms and all participants were followed until March 31, 2009. We used Cox Proportional Hazard models to determine if study arm was independently associated with the development of opportunistic infections (OIs) or death.

**Results:**

We randomized 1211 participants to the three original study arms and 331 surviving participants in the clinical monitoring arm were re-randomized to the CD4-VL and CD4 only arms. At enrolment the median age was 38 years and the median CD4 cell count was 134 cells/μL. Over a median of 5.2 years of follow-up, 37 deaths and 35 new OIs occurred in the VL-CD4 arm patients, 39 deaths and 42 new OIs occurred in CD4-only patients. We did not observe an association between monitoring arm and new OIs or death (AHR =1.19 for CD4-only vs. CD4-VL; 95 % CI 0.82–1.73).

**Conclusion:**

We found no differences in clinical outcomes associated with the addition of quarterly VL monitoring to quarterly CD4 cell count monitoring.

## Background

One of the greatest global public health achievements has been the rapid scaling up of antiretroviral therapy (ART) in resource limited settings over the past decade. This has largely been achieved through the “public health approach” promoted by the World Health Organization (WHO) [1–3]. This approach has involved training a range of different health-care personnel to support delivery and monitoring of ART treatment and care services with the aim of shifting from a centralized, doctor-led model of HIV treatment and care to decentralized models, thus enabling a larger number of people to be initiated and retained in care [3, 4].

The WHO 2003 guidelines for the use of ART initially did not recommend viral load (VL) testing as a necessary component of treatment programs. However, the WHO 2013 guidelines now recommend VL testing as the preferred monitoring approach to diagnose and confirm ART treatment failure in both adults and children. Thus many countries such as Uganda [5] have revised their national guidelines for the provision of ART to recommend VL monitoring as the preferred standard. However, VL testing remains relatively costly and more technologically challenging in comparison to clinical or CD4 cell count monitoring in resource limited settings. Moreover, the WHO scale-up strategy is based on decentralized, integrated delivery of HIV care. However, in rural areas where most patients live, local health facilities generally do not have access to sophisticated laboratories and referral networks for transporting samples to, and receiving results from, centralized laboratories [1, 6]. While there are advantages to providing access to VL testing such as earlier detection of treatment failure and thus a reduced likelihood of developing ART drug resistance, this approach is still debated in resource limited settings [7–11].

The Home-Based AIDS Care (HBAC) project was a 3 arm clinical trial which found that clinical monitoring alone resulted in increased risk of new OIs or death, in comparison to the two other arms where routine laboratory monitoring was available [8]. However, the study found no difference in clinical outcomes between participants who were randomized to VL and CD4 cell count monitoring in comparison to CD4 cell count monitoring, alone after 3 years of follow-up. The only other randomized trial which has directly compared clinical outcomes between patients monitored with VL and CD4 cell counts with those monitored with CD4 cell counts alone, conducted in Thailand found similar results. [12] In, 2007, following the end of the first phase of the HBAC trial, participants who were originally randomized to the clinical monitoring arm were re-randomized to either the VL or the CD4 cell count monitoring arm and all participants were observed for an additional 2 years of follow-up. We now report on the long term clinical outcomes from this study with this additional follow-up time. The objective of this continuation of the HBAC trial was to see if any additional differences emerged with additional follow-up between individuals receiving CD4 cell count monitoring and VL testing in comparison to those individuals who received CD4 cell count testing alone.

## Methods

### Study design

Beginning in May, 2003, we assessed for eligibility for study enrolment of HIV positive adult patients ≥18 years who had registered with The AIDS Support Organization (TASO) - Tororo branch. Enrolment was offered to patients with a CD4 cell count <250 cells/μL or severe HIV disease (defined as WHO stage 3 or 4 or a history of recurrent herpes zoster). Additional enrollment criteria are described elsewhere. [8] We obtained written informed consent from all the study participants that were enrolled in the study. Participants initiated ART with combinations of lamuvidine with either niverapine or efavirenz; and zidovudine or stavudine, In April, 2007, following analysis of the first phase of the study which demonstrated that clinical follow-up only participants were at increased risk for death and/or new opportunistic infections (OIs) [8], these participants were re-randomized to either clinical monitoring and quarterly CD4 cell counts and VL (CD4-VL) or clinical monitoring and quarterly CD4 cell counts only (CD4-only) and all participants were followed until March 31, 2009. Trained lay field workers continued to provide ART to participants at home including collecting data to monitor potential toxicity, morbidity and mortality. However, the frequency of home visits was changed in the second phase of the study over a 4 month period from once per week to once every 2 months. Pre-packaged drugs were replaced by using a storage container, and pill counts were conducted at the study clinic by a pharmacist. Participants were weighed during home visits and these weights and body mass index (BMI) scores were provided to clinicians. After enrolment, no routine clinic visits were scheduled but participants were encouraged to come to the clinic or hospital if they were ill and were transported to the clinic for assessment if they had specifically defined symptoms or severe illness during a home visit.

Monitoring and diagnostic procedures for the occurrence of illness did not differ between study arms. Physicians responsible for patients in the two study arms received laboratory results on a quarterly basis. Participants received daily cotrimoxazole prophylaxis regardless of CD4 cell count except during a five-month cotrimoxazole discontinuation sub-study [13] Participants who had ART treatment failure as per the arm-appropriate definitions below were switched to didanosine, tenofovir, and lopinavir/ritonavir. In the CD4-VL arm, treatment failure was defined as two consecutive viral load measurements ≥500copies/mL occurring more than 6 months after the start of ART. For the CD4-only arm, persistently declining CD4 cell counts on two consecutive measurements was considered to indicate treatment failure. The first response to a worsening trend in CD4 or VL was counselling about adherence to treatment. Study physicians, nurses, counsellors, and other staff met weekly in a case conference to discuss all deaths, opportunistic illnesses, and abnormal laboratory results and approved all regimen changes. A data safety monitoring board reviewed data every 3 months and was asked to reject the null hypothesis of monitoring arm equivalence if the rate of severe morbidity and mortality in any arm exceeded another by three standard errors of the difference (“Haybittle-Peto” rule) [14, 15]. The study received ethics approval from the University of British Columbia, the Uganda Virus Research Institute, and the Institutional Review Board of the United States Center for Disease Control and Prevention and the Uganda National Council for Science and Technology. The trial was registered at ClinicalTrials.gov, Registration number NCT00119093.

### Laboratory procedures

HIV VL was measured with Cobas Amplicor HIV-1 Monitor version 1.5 ultrasensitive assay (Roche, Branchburg, NJ) for baseline measurements, which had a lower limit of detection of 400 copies/mL. Follow-up VL measurements were conducted with the Cobas Taqman (manual extraction) assay, with a lower limit of detection of 50 copies/mL. CD4 cell counts were done with Tri TEST reagents following an in house dual platform protocol and MultiSET and Attractors software with a FAC Scan or FACS Calibur flowcytometer (Becton-Dickinson, Franklin Lakes, NJ). Complete blood counts were provided with CD4 cell counts [6].

### Data analysis

We followed the study participants randomized or re-randomized in the remaining two arms for an additional 2 years up to 21^st^ March 2009. We conducted bivariate analyses of clinical and demographic characteristics of study participants in the remaining two arms. Data were analyzed with SAS 9.0 (SAS Institute, Cary,NC). We used Kaplan-Meier survival curves to graphically compare time to first opportunistic illness (OI) or death after 90 days following ART initiation (or after re-randomization for those who were re-randomized to the CD4-VLor CD4-only arms). Adherence to therapy was calculated using the medication possession ratio. [16] Cox proportional hazards regression models were used to adjust for possible confounding, by age, sex, baseline CD4 cell count, VL and body mass index (BMI). Poisson regression analysis with log link function was used to compare the rates of new opportunistic infections and/or deaths occurring after 90 days following ART initiation (or after re-randomization for those who were re-randomized). Logistic regression models were used to compare the proportions that were switched to second line regimens and proportion that had elevated (≥500 copies/mL) viral loads after 6 months on ART or after re-randomization for those who were re-randomized. Person time for people lost to follow-up or transferred to a different provider was censored at the time of the last home visit at which they received ART.

## Results

**Fig. 1:**
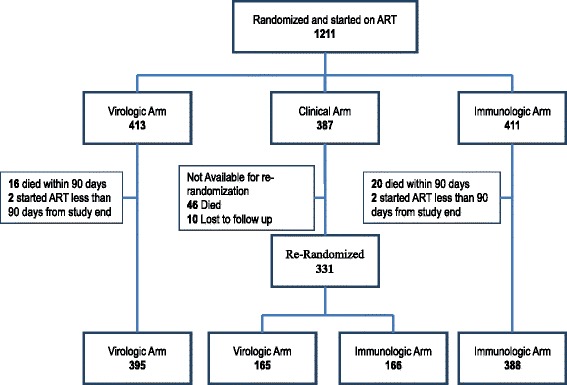
Study profile

A total of 1211 participants were randomized beginning in May 2004 and started on ART in the initial three study arms (413 in VL arm 411 in CD4 cell count arm and 387 in the clinical arm [8]. Overall, 71.8 % of the participants were female, the median age was 38 years (IQR: 32–44) and the median baseline CD4 cell count was 134 cells/mL (IQR: 70–199). In April, 2007, 331 surviving participants in the clinical arm were re-randomized to the VL (165) and CD4 cell count (166) arms (Fig. 1). Demographic and clinical parameters were similar across the two study arms, (Table 1).

**Table 1:** Baseline characteristics of HBAC study participants Tororo and Busia Districts, Uganda, 2003-9, according to type of monitoring: viral load arm

	ALL		Viral load arm		CD4 arm		
Variable	N	%	n	%	n	%	*p*-value
Sex							
F	810	72.7	409	73.0	401	72.4	0.807
M	304	27.3	151	27.0	153	27.6	
Baseline CD4							
<50	197	17.7	97	17.4	100	18.1	0.824
50–200	645	58.1	322	57.7	323	58.5	
>200	268	24.1	139	24.9	129	23.4	
Baseline viral load							
<1000	35	3.2	17	3.1	18	3.3	0.434
1000–9999	47	4.3	28	5.1	19	3.5	
10,000–99,999	311	28.3	148	26.8	163	29.9	
> = 100,000	705	64.2	360	65.1	345	63.3	
Baseline BMI							
<18.5	302	27.8	149	27.4	153	28.1	0.347
18.5–24.9	713	65.5	351	64.5	362	66.5	
25–29.9	53	4.9	32	5.9	21	3.9	
> = 30	20	1.8	12	2.2	8	1.5	

As of April 30, 2009, the median follow-up time for all participants was 5.2 years from the original randomization date and 4.8 years after 90 days on ART (or re-randomization). During follow-up after 90 days on ART (or re-randomization) 37 deaths and 35 new OIs occurred in patients randomized or re-randomized to the CD4-VL arm and 39 deaths. The last median CD4 for VL arm was 560, IQR (324–602) while CD4 arm was 554, 1QR (331–595). We did not find any significant differences between the two arms *p* = 0.986. Forty two (42) new OIs occurred in patients in the CD4 cell count arm. The most common OIs diagnosed among participants were tuberculosis (49 % of OIs), followed by Cryptococcosis (13 %), and Kaposi’s sarcoma (10 %).

**Fig. 2:**
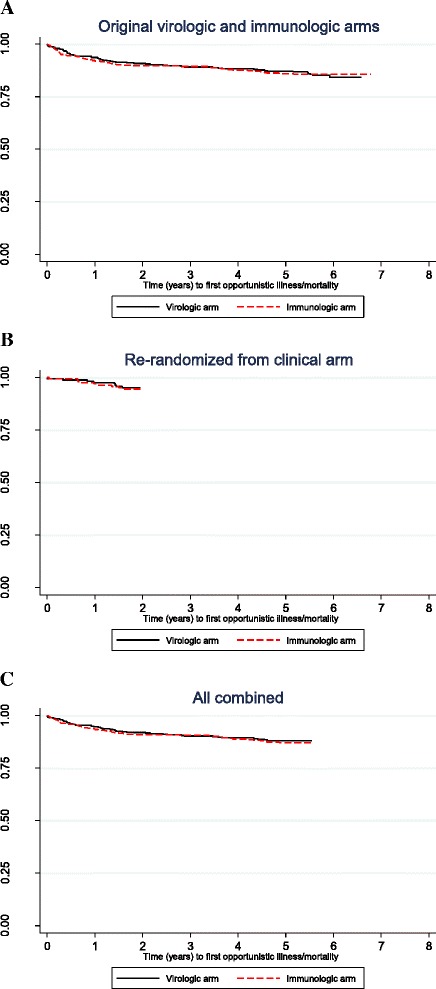
Kaplan-Meier curves of time to first opportunistic illness or death. **a**-**c** Porportion of participants without opportunistic infection/illness/mortality

In a Kaplan-Meier analysis, we found no difference in the time to first event of new OI or mortality between the two monitoring arms (Fig. 2.) rate of 3.0 per 100 person-years in the CD4-VL arm compared to 3.2 per 100 person-years in the CD4 arm; *p* = 0.605 for log-rank test. Adherence was similar across the two study arms with the mean adherence over each visit interval of 99 % in each study arm (*p* = 0.123). In a Cox proportional hazards model with adjustment for baseline age, sex, CD4 cell count, viral load, and BMI, there was no statistically significant difference in the risk of first serious morbidity or death between the CD4 arm and the CD4-VL arm; adjusted hazard ratio [AHR] 1.19, 95 % confidence interval 0.82–1.73) for the CD4 cell count arm in comparison to the CD4-VL arm. We did not find any statistically significant difference between the two arms in terms of mortality (HR =1.12, 95 % CI: 0.70–1.77) (Table 2) or the number of severe morbidity events including death (RR = 1.23, 95 % CI: 0.88–1.71) after adjusting for baseline age, sex, CD4 cell count, viral load, and BMI (data not shown), when analyzed separately.

**Table 2:** Cox proportional hazards regression analysis for time to first morbidity (OI) or mortality event

Arm	# people	# Events	Person years	Rate per 100 person years	Hazard Ratio (95 % CI) ^a^	*p*_value
Original Viral load and CD4 arms						
Viral load arm	395	49	1617.3	3.03	ref.	
CD4 arm	388	53	1607.7	3.30	1.22( 0.82 to 1.84)	0.327
Re-randomized from Clinical arm						
Viral load arm	165	8	313.3	2.55	ref.	
CD4 arm	166	9	314.1	2.87	1.14( 0.42 to 3.10)	0.801
All combined						
Viral load arm	560	57	1930.6	2.95	ref.	
CD4 arm	554	62	1921.9	3.23	1.19( 0.82 to 1.73)	0.368

^a^Adjusted for Age, sex, baseline CD4, Viral load and BMI

During the follow-up, 182 participants had at least one elevated VL measurement (≥500 copies/mL after 6 months or re-randomization for those who were re-randomized; 80 (14.6 %) in the CD4-VL arm, 102 (18.9 %) in the CD4 arm (Table 3). These differences were not statistically significant (Odds ratio = 1.31 for CD4 cell count arm relative to CD4-VL arm, 95 % CI: 0.95–1.83). A total of 54 participants were changed to a second-line regimen (Table 4), 30 (5.3 %) in the CD4-VL arm, and 24 (4.3 %) in the CD4 arm (Table 4). Again these differences were not statistically significant) (OR = 0.76) for the CD4 arm compared to the CD4-VL arm, 95 % CI: 0.44–1.33). Of the 24 individuals in the CD4 arm who were switched to second-line therapy, 11(46 %) were found to have had VLs > 500 copies/mL after 6 months of ART. We noted that a smaller proportion of patients in the CD4-VL arm who ever had two VL results ≥500 copies/mL compared to those in the CD4 monitoring arm (4.6 % vs. 7.5 %). However this difference was not statistically significant (*p* = 0.56). At the close of the study, 92 % of the participants on the CD4 only arm had undetectable viral loads.

**Table 3:** Proportion switched to second line regimen (after re-randomization for those who were re-randomized)

Arm	# people	Number	Percent	Odds Ratio (95 % CI) ^a^	*p*-value
Original Viral load and CD4 arms					
Viral load arm	395	15	3.8	ref.	
CD4 arm	388	13	3.4	0.87( 0.40 to 1.90)	0.727
Re-randomized from Clinical arm					
Viral load arm	165	15	9.1	ref.	
CD4 arm	166	11	6.6	0.70( 0.31 to 1.60)	0.399
All combined					
Viral load arm	562	30	5.3	ref.	
CD4 arm	557	24	4.3	0.76( 0.44 to 1.33)	0.343

^a^Adjusted for Age, sex, baseline CD4, Viral load and BMI

**Table 4:** Proportion switched to second line regimen (after re-randomization for those who were re-randomized)

Arm	# people	Number	Percent	Odds Ratio (95 % CI) ^a^	*p*-value
Original Viral load and CD4 arms					
Viral load arm	395	15	3.8	ref.	
CD4 arm	388	13	3.4	0.87( 0.40 to 1.90)	0.727
Re-randomized from Clinical arm					
Viral load arm	165	15	9.1	ref.	
CD4 arm	166	11	6.6	0.70( 0.31 to 1.60)	0.399
All combined					
Viral load arm	562	30	5.3	ref.	
CD4 arm	557	24	4.3	0.76( 0.44 to 1.33)	0.343

^a^Adjusted for Age, sex, baseline CD4, Viral load and BMI

## Discussion

In this extension of the HBAC study as a two-arm trial, we found no statistically significant differences in clinical outcomes associated with the addition of quarterly VL monitoring to quarterly CD4 cell count monitoring after over 5 years of follow-up. Furthermore, we did not find any differences in terms of the proportion of participants with unsuppressed VL or rate of switching to second-line therapy between these two strategies. Our analysis again suggests that the addition of VL monitoring to CD4 cell count monitoring may not result in improved clinical outcomes for HIV positive patients receiving ART in resource limited settings. This conclusion is the same as that of the original HBAC study and the only other direct comparison of VL and CD4 cell count monitoring, another RCT conducted in Thailand [12, 17]. The latter study reported that a CD4 switching strategy was non-inferior in terms of clinical outcomes among HIV-positive adults, 3 years after beginning ART when compared to a VL -based switching strategy [12]. The authors found that there was also no difference between the strategies in terms of virologic suppression and immune restoration. Importantly, however, even though patients in the CD4 arm spent longer with a high viral load than patients in the VL arm, the emergence of HIV mutants resistant to antiretroviral drugs was similar in the two arms [12]. Unfortunately, we do not have any resistance data in order to make comparisons in this regard.

These findings differ somewhat from the results of an analysis of mortality of patients on ART in Southern Africa from the International epidemiological Databases to Evaluate AIDS in Southern Africa (IeDEA-SA) [8]. Participants from programs which did not have access to VL testing, namely those in Zambia and Malawi reported higher rates of death and loss to follow up, in comparison to participants from South Africa where VL measurement was accessible and readily available. However, it is unlikely that the only differences between these programs related to the provision of VL testing and differences in health care systems and living environments of these patients likely also influenced the differences in outcomes observed. Studies which compared the effect of routine VL testing to the standard of care where VL was used sparingly to adjudicate discrepancies between CD4 and clinical assessments, found that VL monitoring did not reduce death over the first 36 months of ART but did result in earlier ART regimen change [8, 9, 18].

A similar exploratory study by AIDS Clinical Trials Group A5115 that followed up participants for three years and compared a treatment switching strategy based on CD4-only monitoring versus VL thresholds in 21 public hospitals throughout Thailand reported no significant differences in activated or total CD4 cells at study end [19, 20]. Despite the lack of evidence of clinical benefit to support the use of routine VL testing, there may be other reasons to promote increase use of VL testing. Routine monitoring of participants with VL may result in reduction in the time a patient takes a failing regimen and potentially reducing the frequency of developing drug resistant mutations [21]. However, to date, there is very little evidence that the drug resistance mutations which develop while patients are failing their first-line regimens have much effect on the success second-line therapy. A study from Malawi found that virologic responses to a second line regimen among 109 participants with immunologically-defined treatment failure and a measured VL ≥1000 copies/mL was quite good (85 % VL < 400 copies/mL among those with VL measurements at 12 months after switching), although mortality was quite high at 9 %. All patients in this study had viruses with at least one resistance mutation, and 56 % of patients had viruses with thymidine analogue mutations, but the authors did not find an association with these mutations and virologic suppression at one-year after treatment switching [22]. Furthermore, the Thai RCT described above, did not find differences in the accumulation of virologics resistance mutations. More evidence from larger studies are needed to determine whether virologic monitoring can improve outcomes for individuals diagnosed with treatment failure in resource-limited settings. In the interim, designing HIV programmes that maximize retention of patients in the continuum of care and support adherence counselling to treatment should remain the focus of HIV treatment programmes. [23–25] Many programmes in Sub-Saharan Africa have reported a loss to follow up among patients on ART of 20 % or more suggesting potential for improvement [26, 27].

This study has a number of limitations; firstly, the generalizability of our study findings to routine care settings may be limited as participants in this trial were seen and counseled more frequently than is routine in most settings. In the first phase of the HBAC study, participants received weekly home delivery of ART and clinical monitoring by field officers. However, in this phase of the study we extended the interval between home visits to once every 2 months over a 4 month period, in order to reflect standard care models. The intensity of the follow-up likely contributed to the low overall rates of virologic failure and loss to follow-up in comparison to those reported in most other settings. It is also important to note that laboratory evaluations were performed every 3 months, rather than every 6 months that is recommended by WHO. Furthermore, the rates of virological failure in our study were generally lower than most reported programmes from the region, as surveyed in a recent systematic review [27–30].

## Conclusions

In conclusion, we found that clinical outcomes in the first 5 years after ART initiation were not different between participants with access to CD4 testing alone in comparison to those with routine VL and CD4 cell count testing. These data support the continued expansion of access to ART in resource-limited settings, irrespective of the availability of VL testing.

## Abbreviations

TASO: The AIDS Support Organization
HBAC: Home Based AIDS Care
BMI: Body Mass Index
ART: Anti-retroviral therapy
RCT: Randomized Clinical Trial

## References

[1] Gilks CF, Crowley S, Ekpini R, Gove S, Perriens J, Souteyrand Y, Sutherland D (2006). The WHO public-health approach to antiretroviral treatment against HIV in resource-limited settings. Lancet..

[2] World Health Organization guidelines (2013). Towards universal access.

[3] Ivers LC, Kendrick D, Doucette K (2005). Efficacy of antiretroviral therapy programs in resource-poor settings: a meta-analysis of the published literature. Clin Infect Dis.

[4] Jones LE, Perelson AS (2000). Transient viremia, plasma viral load, and reservoir replenishment in HIV-infected patients on antiretroviral therapy. J Acquir Immune Defic Syndr.

[5] Uganda Comprehensive ART guideline. Ministry of Health-Uganda; 2012

[6] George A, Valdez C, Herrera M, Barillas E. Building a supply chain approach for an improved laboratory sample referral network in the Dominican Republic. J Pharm Policy Pract. 2014;7 Suppl 1:P4.

[7] World Health Organization (2003). A public health approach for scaling up antiretroviral (ARV) treatment: a toolkit for programme managers.

[8] Mermin J, Ekwaru JP, Were W, Degerman R, Bunnell R, Kaharuza F (2011). Utility of routine viral load, CD4 cell count, and clinical monitoring among adults with HIV receiving antiretroviral therapy in Uganda: randomised trial. BMJ (Clinical Research Ed).

[9] Saag M, Westfall A, Luhanga D, Mulenga P, Chi B, Arnedo M, Alonso E, et al. A cluster randomized trial of routine vs discretionary viral load monitoring among adults starting ART: Zambia. Seattle (Washington), USA: 19th Conference on Retroviruses and Opportunistic Infections; 2012. Abstract number 87.

[10] Koenig SP, Kuritzkes DR, Hirsch MS, Leandre F, Mukherjee JS (2006). Monitoring HIV treatment in developing countries. BMJ.

[11] Kumarasamy N, Flanigan TP, Mahajan AP, Carpenter CC, Mayer KH (2002). Monitoring HIV treatment in the developing world. Lancet Infect Dis.

[12] Jourdain G, Le Cœur S, Ngo-Giang-Huong N, Traisathit P, Cressey TR, Fregonese F (2013). Switching HIV treatment in adults based on CD4 count versus viral load monitoring: a randomized, non-inferiority trial in Thailand. PLoS Med.

[13] Campbell JD, Moore D, Degerman R, Kaharuza F, Were W, Muramuzi E (2012). HIV-infected ugandan adults taking antiretroviral therapy with CD4 counts 200 cells/mL who discontinue cotrimoxazole prophylaxis have increased risk of malaria and diarrhea. Clin Infect Dis.

[14] Pocock SJ (2005). When (not) to stop a clinical trial for benefit. JAMA.

[15] Peto R, Pike MC, Armitage P, Breslow NE, Cox DR, Howard SV (1976). etal. Design and analysis of randomized clinical trials requiring prolonged observation of each patient. I. Introduction and design. Brit J Cancer.

[16] Laurent C, Kouanfack C, Laborde-Balen G, Aghokeng AF, Mbougua JBT, Boyer S (2011). Monitoring of HIV viral loads, CD4 cell counts, and clinical assessments versus clinical monitoring alone for antiretroviral therapy in rural district hospitals in Cameroon, a randomised non-inferiority trial. Lancet Infect Dis.

[17] Weidle PJ (2006). Adherence to antiretroviral therapy in a home-based AIDS care programme in rural Uganda. Lancet.

[18] Braitstein P, Brinkhof MW, Dabis F, Schechter M, Boulle A, Miotti P (2006). Mortality of HIV-1-infected patients in the first year of antiretroviral therapy: comparison between low-income and high-income countries. Lancet.

[19] Riddler SA, Jiang H, Tenorio A, Huang H, Kuritzkes DR (2007). A randomized study of antiviral medication switch at lower- versus higher-switch thresholds. Antivir Ther.

[20] Tenorio AR, Jiang H, Zheng Y, Bastow B, Kuritzkes DR (2009). Delaying a treatment switch in antiretroviral-treated HIV type 1-infected patients with detectable drug-resistant viremia does not have a profound effect on immune parameters. AIDS Res Hum Retroviruses.

[21] Calmy A, Ford N, Hirschel B, Reynolds SJ, Lynen L, Goemaere E (2007). HIV viral load monitoring in resource-limited regions: optional or necessary?. Clin Infect Dis.

[22] Hosseinipour MC, Kumwenda JJ, Weigel R, Brown LB, Mzinganjira D, Mhango B (2010). Second-line treatment in the Malawi antiretroviral programme: high early mortality, but good outcomes in survivors, despite extensive drug resistance at baseline. HIV Med.

[23] Stringer JS, Zulu I, Levy J, Stringer EM, Mwango A, Chi BH (2006). Rapid scale-up of antiretroviral therapy at primary care sites in Zambia: feasibility and early outcomes. JAMA.

[24] Wools-Kaloustian K, Kimaiyo S, Diero L, Siika A, Sidle J, Yiannoutsos CT (2006). Viability and effectiveness of large-scale HIV treatment initiatives in sub-Saharan Africa: experience from western Kenya. AIDS.

[25] Mugyenyi P, Walker AS, Hakim J, Munderi P, Gibb DM, Kityo C (2010). Routine versus clinically driven laboratory monitoring of HIV antiretroviral therapy in Africa (DART). Lancet.

[26] Phillips AN, Pillay D, Miners AH, Bennett DE, Gilks CF, Lundgren JD (2008). Outcomes from monitoring of patients on antiretroviral therapy in resource-limited settings with viral load, CD4 cell count, or clinical observation alone: a computer simulation model. Lancet.

[27] Brigido L, Rodrigues R, Casseb J, Custodio RM, Fonseca LA, Sanchez M (2005). CD4 + T-cell recovery and clinical outcome in HIV-1-infected patients exposed to multiple antiretroviral regimens: partial control of viremia is associated with favourable outcome. AIDS Patient Care STDS.

[28] Freya R, Barbara T, Faustino L, Tom D, Daniel R, Marc B (2014). A Qualitative Assessment of a Community Antiretroviral Therapy Group Model in Tete, Mozambique. PLoS One.

[29] Francois D, Eric B, Paula B, Paolo M, Martin Brinkhof WG, Martin S (2005). Cohort Profile: Antiretroviral Therapy in Lower Income Countries (ART-LINC): international collaboration of treatment cohorts. Int J Epidemiol.

[30] Hardon AP, Akurut D, Comoro C, Ekezie C, Irunde HF (2007). Hunger, waiting time and transport costs: time to confront challenges to ART adherence in Africa. AIDS Care.

